# Development and Validation of Extracorporeal Membrane Oxygenation Mortality-Risk Models for Congenital Diaphragmatic Hernia

**DOI:** 10.1097/MAT.0000000000000716

**Published:** 2018-10-31

**Authors:** Yigit S. Guner, Danh V. Nguyen, Lishi Zhang, Yanjun Chen, Matthew T. Harting, Peter Rycus, Ryan Barbaro, Matteo Di Nardo, Thomas V. Brogan, John P. Cleary, Peter T. Yu

**Affiliations:** From the * Division of Pediatric Surgery, Children’s Hospital of Orange County, California and Department of Surgery, University of California Irvine Medical Center, Orange, California; † Department of Medicine, University of California Irvine, Orange, California; ‡ Institute for Clinical and Translational Science, University of California, Irvine, California; § Department of Pediatric Surgery, University of Texas McGovern Medical School and Children’s Memorial Hermann Hospital, Houston, TX; ¶ Extracorporeal Life Support Organization Ann Arbor, Michigan; ‖ Department of Pediatrics, and Child Health Evaluation and Research (CHEAR) Unit, University of Michigan, Ann Arbor, Michigan; # Ospedale Pediatrico Bambino Gesu, Rome, Italy; ** Department of Pediatrics, University of Washington, Seattle Children’s Hospital, Washington.

**Keywords:** ECMO, CDH, mortality risk, risk score

## Abstract

The purpose of our study was to develop and validate extracorporeal membrane oxygenation (ECMO)–specific mortality risk models for congenital diaphragmatic hernia (CDH). We utilized the data from the Extracorporeal Life Support Organization Registry (2000–2015). Prediction models were developed using multivariable logistic regression. We identified 4,374 neonates with CDH with an overall mortality of 52%. Predictive discrimination (*C* statistic) for pre-ECMO mortality model was *C* = 0.65 (95% confidence interval, 0.62–0.68). Within the highest risk group, based on the pre-ECMO risk score, mortality was 87% and 75% in the training and validation data sets, respectively. The pre-ECMO risk score included pre-ECMO ventilator settings, pH, prior diaphragmatic hernia repair, critical congenital heart disease, perinatal infection, and demographics. For the on-ECMO model, mortality prediction improved substantially: *C* = 0.73 (95% confidence interval, 0.71–0.76) with the addition of on-ECMO–associated complications. Within the highest risk group, defined by the on-ECMO risk score, mortality was 90% and 86% in the training and validation data sets, respectively. Mortality among neonates with CDH needing ECMO can be reliably predicted with validated clinical variables identified in this study. ECMO-specific mortality prediction tools can allow risk stratification to be used in research and quality improvement efforts, as well as with caution for individual case management.

Despite advances in neonatal care, the mortality rate of infants with congenital diaphragmatic hernia (CDH) treated with extracorporeal membrane oxygenation (CDH-ECMO population) has remained unchanged.^1,2^ Accurate discrimination of disease severity in the CDH-ECMO population is required to test and improve current treatment strategies. Mortality risk prediction equations developed for the general CDH population do not discriminate well within the ECMO cohort.^3–7^ In 2008, Haricharan *et al*.^8^ reported a CDH-ECMO mortality prediction score using Extracorporeal Life Support Organization (ELSO) Registry data. The Haricharan score included demographic variables, on-ECMO variables, and support duration >15 days, modeled together as an on-ECMO mortality risk model.^8^ The Haricharan model has not been externally validated and has not been adopted as a clinical or a research tool. Additional data have since been collected by the ELSO Registry to develop more robust risk models that predict mortality separately before ECMO and during ECMO. More recently, neonatal risk estimate score for children using extracorporeal respiratory support (Neo-RESCUERS) and Pittsburgh index for pre-ECMO risk (PIPER) mortality prediction models were developed inclusive of all neonatal conditions receiving respiratory ECMO.^1,9^ However, Neo-RESCUERs and PIPER were not specifically developed for CDH nor validated specifically in a CDH-specific data set. It is well established that CDH has the greatest mortality rate of all other neonatal conditions requiring respiratory ECMO. Furthermore, treatment of CDH with ECMO is inherently more complex given the anatomic complexities associated with herniation of intra-abdominal contents to the thorax and the surgical treatment that is needed to repair the diaphragmatic defect. For all those reasons, we sought to develop and validate ECMO mortality risk models specific for the CDH population.

Given that ECMO is an invasive treatment, ECMO mortality prediction models are most informative when designed to provide mortality risk before exposure to ECMO and then at any time point while the patient is receiving extracorporeal life support. We hypothesized that two separate models would prove to be most relevant in predicting mortality risk associated with ECMO in the CDH population: 1) before initiation of ECMO, and 2) during the course of ECMO. Two separate models were developed to analyze the initial risk mortality associated with ECMO and the risk while on-ECMO. We took into account the possible contributions of pre-ECMO rescue therapies, anatomic variations of CDH, timing of diaphragm repair, comorbidities, ECMO complications, and length of ECMO. Although bedside usefulness of such models should never replace clinical acumen, mortality risk models are useful when analyzing and benchmarking patient outcomes and assessing the value of programmatic changes. The ability to benchmark against known pre-ECMO risk and demonstrating a lower on-ECMO risk is the ultimate goal of proving good ECMO therapy. This is why we believed it was critical to provide risk models that sought to independently predict risk of mortality before and during ECMO.

## METHODS

### Data Source and Cohort

The Children’s Hospital Orange County institutional review board approved this study (No. 150969). We queried the ELSO Registry data for neonates whose primary diagnosis was CDH from 2000 to 2015. We omitted data from before 2000 to limit the data to the most current treatment practices. We searched ELSO Registry for secondary international classification of diseases, ninth revision (ICD-9) diagnoses codes to establish dichotomous variables to identify complications/comorbidities. Candidate predictors evaluated for models were selected based on clinical considerations and previous studies.^2,3,6–8,10–16^

### Candidate Variables

For the pre-ECMO model, we considered the following demographic variables, including gender, pre-ECMO weight, race, gestational age (GA), post-GA, 5 min Apgar, side of CDH, prenatal diagnosis of CDH, CDH repair before ECMO, handbagging, and pre-ECMO arrest; blood gas/ventilator variables included pH, pCO_2_ and pO_2_, mean airway pressure (MAP), oxygenation index; pre-ECMO therapies included inotropes, bicarbonate/tromethamine, inhaled nitric oxide, surfactant, neuromuscular blockers, milrinone, sildenafil and steroids; comorbidity variables included critical congenital heart disease,^17,18^ multiple congenital anomalies, chromosomal anomalies, perinatal infection, and air leak.

For the on-ECMO model, we identified additional variables including repair of diaphragmatic hernia on-ECMO and ECMO duration, ECMO mode (venoarterial and venovenous)^2^ and pump type, and comorbidities including peritonitis, sepsis, and airleak syndrome. We grouped complications by systems or used them individually depending on clinical relevance: mechanical, hemorrhagic (excluding pulmonary hemorrhage which was used independently), cardiac (including stun, tamponade, and need for cardiopulmonary resuscitation (CPR), infectious (positive cultures and white blood cell < 1500), and endocrine complications (glucose < 40 and >240) were grouped. Neurologic complications were divided into seizures (clinical and electrographic) and severe neurologic complications (central nervous system (CNS) hemorrhage, infarct, intraventricular hemorrhage grade 3 and 4); renal complications were separated into two elevated creatinine groups (1.5–3 and >3) and dialysis (hemofiltration, CAVHD).

### Exclusion Criteria and Missing Values

We excluded patients with missing sex and ECMO mode. We reported results based on mean imputation to address missing values in 5 min Apgar, pCO_2_, pO_2_, OI, and duration of ECMO. Sensitivity analyses were conducted using multiple imputation (10 imputations) as well as on complete data. Missing values in pre-ECMO weight (2.4%) were imputed based on a regression model of nonmissing weight with birth weight (BW) and age (days) as independent variables. Similarly, missing values in GA (4.5%) were imputed based on decile groups of BW. The Henderson–Hasselbalch equation was used to calculate missing pH (3.5%), given known HCO_3_ and pCO_2_. MAP (10.2%) was imputed based on a clinical formula as a function of peak inspiratory pressure, respiratory rate, and positive end expiratory pressure. OI was calculated as OI = [(fio_2_×MAP)/pO_2_)], and missing values (10.2 %) were obtained using mean imputation.

### Statistical Methods

The outcome of the prediction models was inpatient mortality during or after ECMO. Patient characteristics were provided as means ± standard deviation (SD) or proportions for continuous and categorical variables, respectively. Prediction scores were developed separately for pre-ECMO and on-ECMO models. The cohort (N = 4,374) was randomly divided into a two-thirds training/development set (N_d_ = 2,912) and a one-third test/validation set (N_v_ = 1,462). Prediction models were developed using multivariable logistic regression models. The final models with reduced number of predictors were obtained using backward selection based on the Akaike information criterion.^19^ We estimated a linear shrinkage factor (γ) using the bootstrap method (with 2000 bootstrap replications) applied to the development data set to assess potential model overfitting (optimism).^19–22^ The shrinkage factor γ was used to adjust the final prediction models to correct for model over-optimism. Overall model calibration was assessed by the Hosmer–Lemeshow goodness-of-fit test and examination of calibration plots.

Model predictive performance or discrimination was assessed using the *C* statistic (area under the receiver operating characteristic curve (ROC) curve) on the one-third validation set. The final prediction models (pre- or on-ECMO) were used to estimate the predicted probabilities of death given the characteristics of a new patient given their calibrated risk score (RS), RS = γXβ, where X represents patient variables, β are the final model coefficients, and γ is the shrinkage factor. The predicted probability for a new patient was 1/(1 + *e*^−RS^). Furthermore, we explored five clinical risk groups (RGs) based on percentiles of the RS (lowest 5%, 5%–25%, 25%–75%, 75%–95%, and highest 5%). The observed mortality in each of the five RGs was assessed in the validation set. Finally, we examined summary statistics of the predictor variables in the five clinical RGs to further understand and identify salient features of patients in each RG. Analyses were performed in R version 3.22 using library RMS and SAS version 9.3.

## RESULTS

### Baseline Characteristics

Baseline characteristics of the cohort are provided in **Table 1**. The majority were male and white race. The mean pre-ECMO weight was 3.07 ± 0.52 kg and GA was 38.1 ± 1.71. Average age at cannulation exceeded 2 days, and ECMO duration was nearly 12 days. Overall, mortality reached 52.4% (2291 deaths). Summary of all predictor variables, including pre-ECMO blood gas, ventilator settings, rescue therapies, comorbidities, along with ECMO modality and pump type and ECMO comorbidities/complications, are detailed in **Table 1**.

**Table 1.**
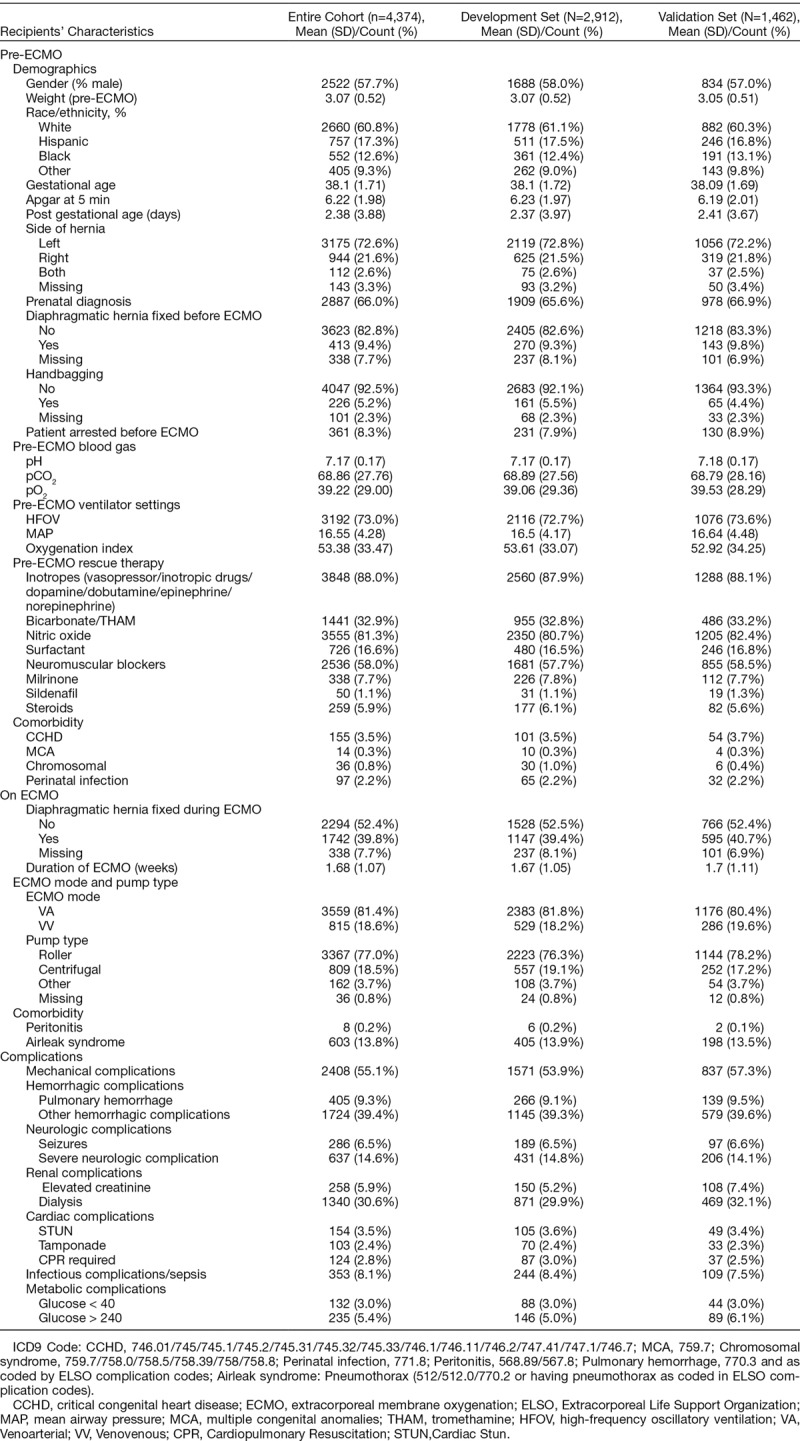
Predictor Variables, Including Baseline Patient Characteristics

### Development of the Prediction Models

We developed two mortality prediction models/scores: pre- and on-ECMO for CDH. The coefficient estimates for the pre-ECMO model are shown in **Table 2**. Lower weight, Apgar score, pH, MAP, bilateral diaphragmatic hernia, repair on-ECMO, prenatal diagnosis, handbagging, pre-ECMO arrest, HFOV, concomitant CCHD, and presence of perinatal infection were associated with increased odds of mortality. Right-sided hernia was associated with decreased odds of mortality. **Table 3** depicts the final prediction model coefficients for the on-ECMO model. In addition to the above significant predictors in the pre-ECMO model, we found that longer ECMO duration, use of inhaled nitric oxide, the presence of multiple congenital anomalies or airleak syndrome, other hemorrhagic complications, severe neurologic complications, tamponade, infectious complications, elevated creatinine/dialysis, and CPR were also associated with increased mortality risk.

**Table 2.**
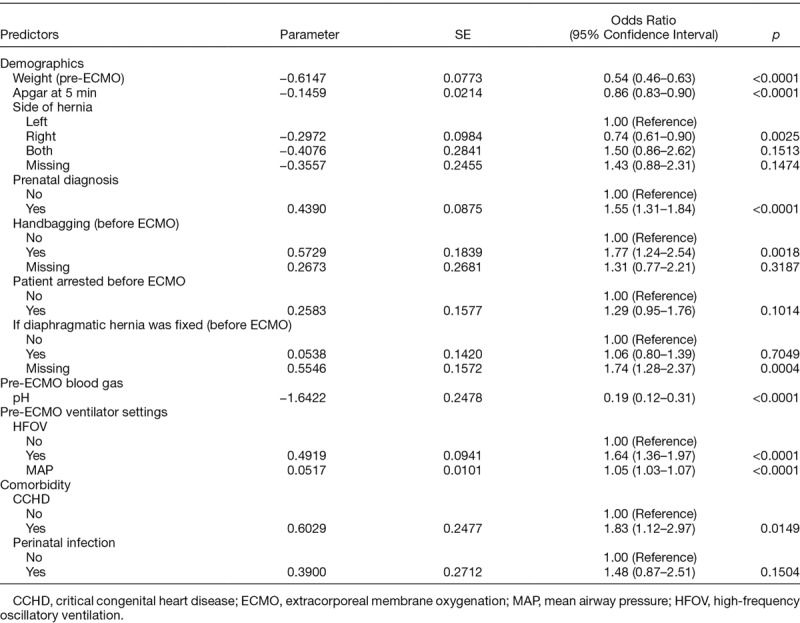
Pre-ECMO Model for Predicting Mortality

**Table 3.**
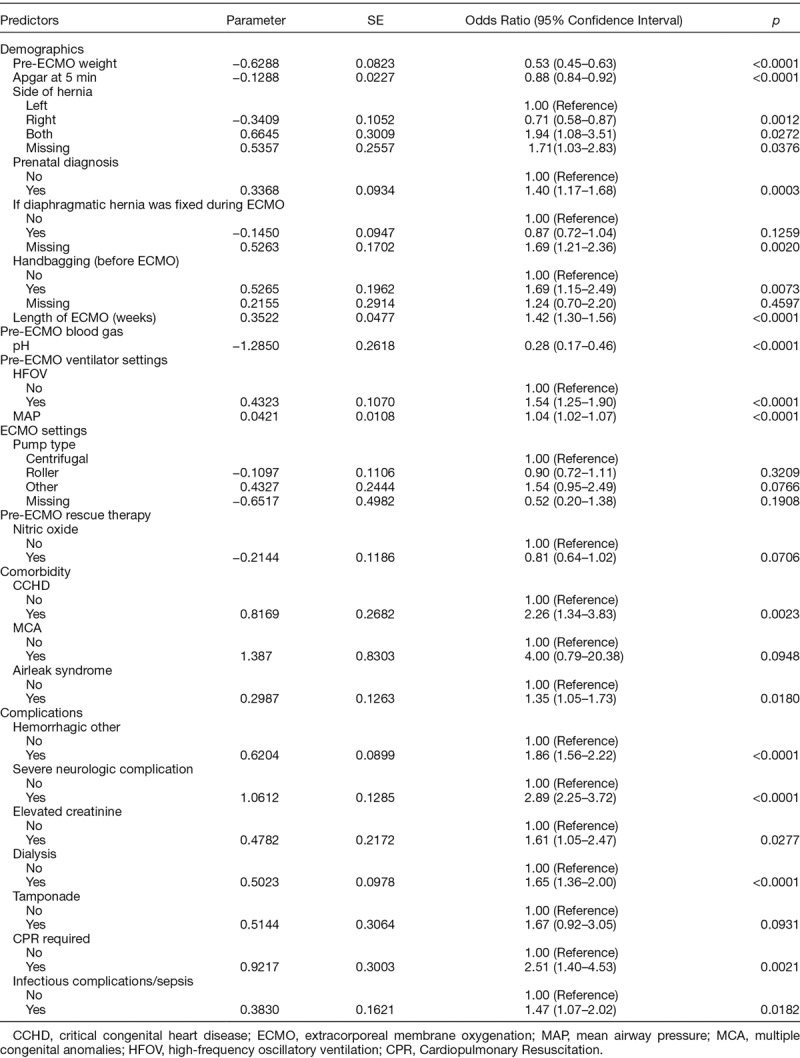
On-ECMO Model for Predicting Mortality

### Internal Validation

Model predictive discrimination was assessed on the validation dataset (n=1,462). For the pre-ECMO model, *C* statistic was 0.65 (95% confidence interval [CI], 0.62–0.68). Applying the development data set used in this study, the Neo-RESCUERS equation discrimination was lower (*C* = 0.59, 95% CI, 0.56–0.62). Note that, the 95% CIs only overlap at the end point of 0.62 (upper limit for Neo-RESCUERS and lower limit for our pre-ECMO score). Thus, there is substantial improvement relative to the Neo-RESCUERS score. The results suggest that the pre-ECMO score of our study discriminates better as it specifically focuses on the CDH population. Revalidation of the PIPER equation in our CDH-specific training data set resulted in *C* statistic (*C* = 0.60; 95% CI, 0.57–0.63). Similarly, compared to the PIPER score there is little overlap in the CIs (upper limit of 0.63 for PIPER and lower limit of 0.62 our pre-ECMO score).

For the on-ECMO model, improved performance to discriminate mortality was observed, given a higher *C* statistic of 0.73 (95% CI, 0.71–0.76). Based on the final variables selected by the model, complications during the ECMO procedure as well as some ECMO-related variables played a significant role in predicting mortality, resulting in a higher *C*-statistic score compared with pre-ECMO model, as expected. When revalidated using the same development data set for this study, the *C* statistic for the Haricharan model was 0.67 (95% CI, 0.68–0.71). Again, note that CI overlap is only at the end point (0.71 is upper limit for Haricharan, and it is the lower upper limit for our on-ECMO model), thus demonstrating better discrimination with our model. Similarly, when PIPER+ was revalidated in our development data set and had decreased discrimination accuracy (*C* = 0.70, 95% CI, 0.67–0.73). There was only a slight overlap in the CIs (upper limit of 0.73 for PIPER+ and lower limit of 0.71 for our on-ECMO score).

**Figure 1.**
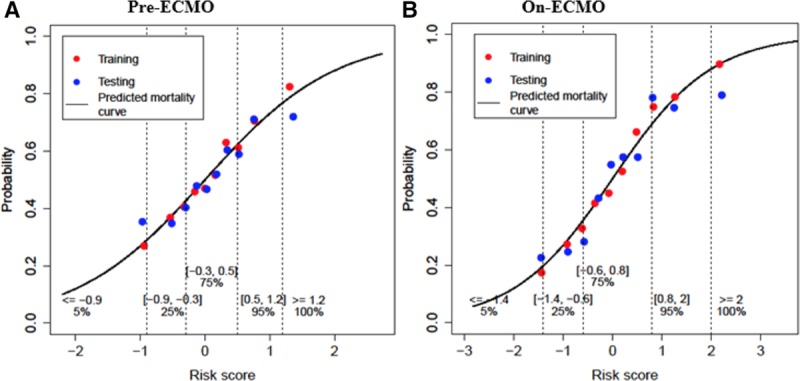
Predicted probability of mortality for pre-ECMO model (**A**) and on-ECMO (**B**) as a function of risk score. Red and blue dots represent observed mortality in groups based on decile of the risk score in development and validation set, respectively. Vertical dashed lines indicate the cutoff for five defined risk groups. ECMO, extracorporeal membrane oxygenation.

A Hosmer–Lemeshow test was used to test the calibration: the χ^2^ goodness-of-fit statistic was 5.85 (*p* = 0.67) for the pre-ECMO model and 6.26 (*p* = 0.62) for the on-ECMO model, indicating that both prediction models fit (*p* < 0.05). The shrinkage factor γ based on 2000 bootstraps is 0.89 (95% CI, 0.79–1.00) in the pre-ECMO and 0.90 (95% CI, 0.83–0.99) in the on-ECMO model, which was used to adjust the final prediction models. **Figure 1** shows the predicted mortality as a function of (A) pre-ECMO and (B) on-ECMO RSs (smooth curve) along with the actual observed mortality rate by decile of the RS in the development and validation data sets. The close agreement between observed and predicted mortality in **Figure 1** provide additional validation of the goodness-of-fit of the prediction models.

To assess the robustness of these models to missing data, we refitted the models using only complete data, as well as multiple imputation using 10 imputed data sets. The estimates of coefficients were quite similar for the models in both sensitivity analyses (results not shown). For the pre-ECMO model, the *C* statistic was 0.65 (95% CI, 0.62–0.68) on complete data analysis and 0.64 (95% CI, 0.61–0.68) on multiple imputation analysis. For the on-ECMO model, *C* statistics were both 0.73 (95% CI, 0.70–0.76), which matched the main results presented above based on mean imputation.

### Exploration of Clinical RGs and Patient Features Within RGs

**Figure 2.**
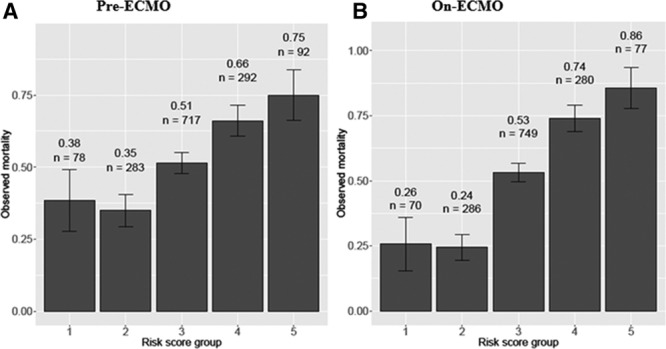
Observed rate of mortality in the validation cohort according to the five risk score groups; n = number of patients in each of the five risk groups ((1) lowest 5%, (2) 5%–25%, (3) 25%–75%, (4) 75%–95%, and (5) highest 5% of the RS for both pre- and on-ECMO models). Error bar is the 95% confidence interval of death rate. ECMO, extracorporeal membrane oxygenation. A: Depicts risk groups per the pre-ECMO risk score. B: Depicts the risk groups per the On-ECMO risk score.

We examined predicted mortality in five clinical RGs, defined a priori based on percentiles of the RS, as (1) lowest 5%, (2) 5%–25%, (3) 25%–75%, (4) 75%–95%, and (5) highest 5% of the RS for both pre- and on-ECMO models. In pre- and on-ECMO data sets, RSs detected 2–4 fold differences in mortality. For the pre-ECMO model, groups 1–5 corresponded to RS ≤ −0.9, (0.9, −0.3), (−0.3, 0.5), (0.5, 1.2) and RS > 1.2, respectively (**Figure 2A**). The observed mortality rates in validation data set for groups 1–5 were 38%, 35%, 51%, 66%, and 75%, respectively (**Figure 2A**); thus, mortality for neonates with RS in the 5th to 25th percentile appeared to be the same as those in the lowest 5% of the RS, while mortality increased for those with RS greater than the 25th percentile. This suggested combining groups 1 and 2 into a single lower RG. Similarly, we defined the RGs for on-ECMO model based on the same percentile groups as the pre-ECMO model above; here the five groups corresponded to on-ECMO RS ≤ −1.4, (1.4, −0.6), (-−.6, 0.8), (0.8, 2.0), and > 2.0 (**Figure 2B**). The observed mortality rates in the validation set corresponding to the five RGs were 26%, 24%, 53%, 74%, and 86%, respectively (**Figure 2B**).

Finally, we illustrate how the models predict pre-ECMO and on-ECMO mortality for several “new” (potential) neonates. **Table 4** shows the predicted probability of death for 3 distinct neonates (patients 1A–1C) pre-ECMO and on-ECMO (patients 2A–2C) with the RGs depicted. Overall, these demonstrate how the models estimate mortality based on each patient characteristics within the ELSO Registry data elements.

**Table 4.**
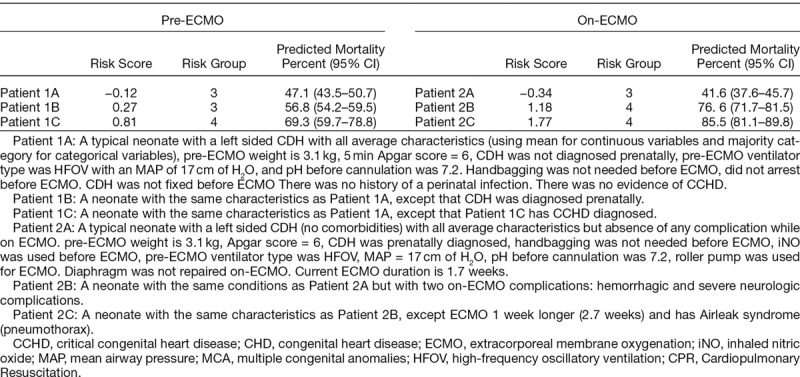
Predicted Pre- and On-ECMO Probability of Death (%) for Potential Neonatal Characteristics

## DISCUSSION

The primary objective of our study was to develop and validate mortality risk prediction models specifically for the CDH-ECMO population. We have noted that prior^1,9^ pre-ECMO risk models can overestimate mortality if the presence or absence of on ECMO complications are not considered. And, we wanted to be able to compare initial mortality risk to risk during ECMO to allow for assessment of quality of ECMO care provided. This was the reason for choosing to develop two independent models to estimate mortality risk for the CDH-ECMO population. Our models were divided into distinct clinical time points where this information could be most useful: pre- and on-ECMO. We believe that the risk models presented in our study use clinically relevant predictor variables and enable clinicians to ask questions such as: “What is the mortality risk of a low BW infant with a right-sided diaphragmatic defect if were to be treated with ECMO?” and “How does the mortality risk change after 2 weeks of ECMO with severe intraventricular hemorrhage and/or other complications?”. The most suitable application of these models is to properly risk-stratify infants, retrospectively, accounting for all available clinical data for research and quality improvement.

Parallels exist between the pre-ECMO model developed in this study and previous risk models developed for the general CDH population, which combined ECMO and non-ECMO data. The CDH Study Group (CDHSG) score was based on 5 min Apgar and BW^3^. The Wilford Hall/Santa Rosa prediction equation (WHSR = highest PaO_2_ − highest PCO_2_) was developed next.^4^ Hoffman *et al*.^5^ later showed that neither of these scores were adequately discriminatory when specifically revalidated within the ECMO population. More recently, Brindle *et al*.^6^ developed a simple CDH scoring equation based on low BW (<1.5 kg), Apgar scores, severe pulmonary hypertension, critical congenital heart disease, and chromosomal anomalies. Unfortunately, the Brindle score is not applicable to the ECMO population as BW < 1.5 kg is not feasible for ECMO. Kays *et al*.^7^ also reported a CDH mortality prediction model, derived from a single institution experience (n = 172), based on CDHSG score, 1 min Apgar, and first pH. Revalidation of the Kays equation with our data set is not possible as first pH is not coded as a variable within ELSO registry data. We revalidated and compared the Neo-RESCUERs and PIPER equations in our data set; based on *C* statistic, our pre-ECMO risk model provided improved prediction.

We next compared the on-ECMO model to previously developed mortality risk models. The first study for comparison is by Seetharamaiah *et al*.,^14^ who determined from CDHSG data (1995–2005) predictors associated with survival in the CDH-ECMO population that underwent CDH repair. Seetharamaiah *et al*.^14^ identified GA, BW, prenatal diagnosis, length of ECMO, and patch repair as survival indicators. We cannot comparatively revalidate the Seetharamaiah predictors with ELSO data, as the ELSO Registry does not record whether repair with patch was used. Our on-ECMO score can be directly compared with the Haricharan’s equation. When revalidated using the same development data set for this study, the *C* statistic for the Haricharan model and PIPER+ had lower discrimination accuracy, thus, demonstrating better discrimination with our model. This improved discrimination can be attributed to expanded data points and model selection methods used in this study.^20^

We made several observations after examination of the RGs for the pre- and on-ECMO models. For both models, analysis of RG distributions in the two lowest RGs (1 and 2) does not differ significantly with similar neonatal characteristics. Also, the pattern of increasing mortality as a function of increasing RGs is similar for both models. Several subtle differences exist between the two models in the distribution of RGs. First, for the pre-ECMO model, mortality estimate is greater by about 10% for groups 1 and 2 (low risk) compared with the same RGs of the on-ECMO model. Second, the two highest RGs of the on-ECMO model have observed mortality about 10% higher than the corresponding RGs for the pre-ECMO model. This improved discrimination of mortality between lower and higher RGs is attributed to additional information (predictor variables) for the on-ECMO model. It is also critical to point out that the pre-ECMO model demonstrated here and by previous studies can overestimate risk in absence of length of ECMO and on-ECMO complications. This point becomes important as CDH patients represent the largest group of neonatal respiratory failure patients experiencing prolonged ECMO courses.^23^ Therefore, the pre-ECMO model provides an average risk of mortality assuming some patients will develop certain complications and have prolonged ECMO runs. This can be helpful as the interplay between the RSs provide a means to address, pinpoint, and improve ECMO care. The on-ECMO model, therefore, is a better prediction tool to estimate mortality risk, assuming those clinical parameters are known.

Clinicians should be very cautious in the application of this or other RSs at the bedside. We specifically discourage clinicians from withholding ECMO for neonates based on high RSs, as survival in the highest RG is 35% and the RS should never come before clinical acumen. Including on-ECMO data may help teams and families understand why support is continuing or occasionally with explaining why discontinuation of support is being considered. Although ideally clinical risk indexes can be used at the bedside, the RSs developed in this study, as well as all other ECMO mortality RSs mentioned above, are best suited for analyzing groups of patients as opposed to the individual neonate. The ECMO risk equations can be used similar to the pediatric American College of Surgeons National Surgical Quality Improvement Program risk equation to provide risk-stratified outcome information to institutions on a periodic basis on CDH infants requiring ECMO.^24^ Furthermore, the scores can be used to analyze patients for quality improvement purposes within the same organization. Future iterations of the risk equation may include local institutional adjustments, as predicted outcomes may be different, for instance, at ECMO centers of excellence or high volume centers, which can only be identified with proper risk adjustment methods, and we believe that the mortality risk equations developed in this study provide the statistically most accurate means to provide such information for the CDH population. Finally, the risk equations can be used for multiple research questions and comparative analyses.

Although our findings add to existing data on CDH-ECMO risk prediction, limitations exist. Similar to most retrospective studies, our study may include potential coding errors and/or missing data. Precise indications for employing ECMO are not standardized across institutions, neither are ECMO care protocols. There are variations in treatment of CDH before ECMO and during ECMO and timing of diaphragm repair across institutions. The clinical variability introduces unmeasurable heterogeneity and randomness, which may affect outcomes. Another limitation was the inability to know the contribution of ECMO to mortality, as the ELSO Registry only includes data for ECMO patients. Therefore, the pre-ECMO risk model should only be calculated in infants who will be treated with ECMO or where ECMO is strongly considered. As is inherent in many databases, the general issue of selection bias is a major limitation, and for the ELSO Registry, there is a selection bias in that it contains patients for whom ECMO has been selected as therapy. Thus, ELSO data reflect the outcomes of patients with CDH for whom ECMO was chosen. Therefore, our prediction model is not a general prediction model of outcome for all CDH patients to be used to decide whether to select ECMO as a therapy or not. Finally, we note that potential candidate predictor variables are limited by what is available in ELSO.

The models developed in this study account for whether or not prenatal diagnosis was established. Important potential information on prenatal prognosticators including lung–head ratio, MRI lung volumes and liver up or down were not available as data elements in the ELSO Registry. Had they been available, these could have potentially improved prediction performance, only in those patients who are prenatally diagnosed. Given, however, prenatal measurements such as lung–head ratio or MRI lung volumes are highly variable on GA, as well as center, and standardization is lacking such that these could be reported to a central registry with accuracy, that is, different centers measure slightly different versions of these anatomic indexes at different gestation ages and not all reports were observed to expected values.^25^ Furthermore, there are more centers who provide ECMO than centers who have established fetal centers. Future studies could be aimed at standardizing fetal prognostication and comparatively validating prenatal risk assessment to postnatal risk assessment methods.

In conclusion, we have developed risk models for CDH that allow mortality risk prediction just before and during ECMO using data reported to the ELSO Registry. The equations developed in this study improve upon previous efforts to define risk in the CDH-ECMO population with increased statistical accuracy. At present, our scores can serve as excellent research tools and for benchmarking outcomes amongst different centers. The ability to assess outcome risk systematically and objectively may allow for a greater patient-centered decision making process and improve the care of these high RGs of neonates. Online calculators for both pre- and on-ECMO models are freely accessible at www.choc.org/ecmocalc, where the predicted mortality, confidence interval, and RG can be calculated rapidly and efficiently.
